# A Novel H2S-releasing Amino-Bisphosphonate which combines bone anti-catabolic and anabolic functions

**DOI:** 10.1038/s41598-017-11608-z

**Published:** 2017-09-20

**Authors:** Simona Rapposelli, Laura Gambari, Maria Digiacomo, Valentina Citi, Gina Lisignoli, Cristina Manferdini, Vincenzo Calderone, Francesco Grassi

**Affiliations:** 1 Dipartimento di Farmacia, Università di Pisa, Via Bonanno 6, I-56126 Pisa, Italy; 2 Laboratorio RAMSES, Istituto Ortopedico Rizzoli, Via di Barbiano 1/10, 40136 Bologna, Italy; 3 S.C. Laboratorio di Immunoreumatologia e Rigenerazione Tissutale, Istituto Ortopedico Rizzoli, Via di Barbiano 1/10, 40136 Bologna, Italy

## Abstract

Bisphosphonates (BPs) are the first-line treatment of bone loss resulting from various pathological conditions. Due to their high affinity to bone they have been used to develop conjugates with pro-anabolic or anti-catabolic drugs. We recently demontrated that hydrogen sulfide (H_2_S), promotes osteogenesis and inhibits osteoclast differentiation. Here we developed an innovative molecule, named DM-22, obtained from the combination of alendronate (AL) and the H_2_S-releasing moiety aryl-isothiocyanate. DM-22 and AL were assayed *in vitro* in the concentration range 1-33 μM for effects on viability and function of human osteoclasts (h-OCs) and mesenchymal stromal cells (h-MSCs) undergoing osteogenic differentiation. Amperometric measures revealed that DM-22 releases H_2_S at a slow rate with a thiol-dependent mechanism. DM-22 significantly inhibited h-OCs differentiation and function, maintaining a residual h-OCs viability even at the high dose of 33 μM. Contrary to AL, in h-MSCs DM-22 did not induce cytotoxicity as revealed by LDH assay, significantly stimulated mineralization as measured by Alizarin Red staining and increased mRNA expression of Collagen I as compared to control cultures. In conclusion, DM-22 is a new BP which inhibits h-OCs function and stimulate osteogenic differentiation of h-MSCs, without cytotoxicity. DM-22 is an ideal candidate for a novel family of osteoanabolic drugs.

## Introduction

A progressive decline in bone strength is a common consequence of aging and several bone-wasting diseases in humans. Osteoporosis, the most prevalent cause of bone fragility, affects 1 in 2 women and 1 in 5 men age 50 and above and causes up to 9 million fractures per year worldwide^1,2^. Among pharmacological therapies, bisphosphonates (BPs) are the first-line and the most prescribed treatment for a number of diseases leading to abnormal bone turnover, including osteoporosis^1^. Key to the mechanism of action of BPs is the high affinity of these molecules for the mineralized bone matrix, which arises from the P-C-P backbone structure^3,4^. Once BPs bind hydroxyapatite within the bone matrix, the acidic pH caused by osteoclasts (OCs) resorption induces their dissociation from the mineral surface and the subsequent internalization within the OCs^5^. Owing to their strong affinity for the bony mineralized matrix, BPs have been broadly developed both as a powerful drugs for bone metabolism and as a carrier to achieve a targeted delivery of bone active molecules^6,7^. Among BPs, amino BPs (N-BPs) were found to be far more potent than simple BPs at inhibiting bone resorption^8^. N-BPs inhibit the mevalonate pathway by targeting the farnesyl diphosphate synthase (FPPS)^9^, leading to the accumulation of unprenilated GTPases in the cytoplasm, which results in toxicity and cell death^9,10^.

Clinical studies have conclusively showed that long-term use (up to 10 years) of BPs is associated to a good safety profile and to a significant reduction in the risk of vertebral, non-vertebral and hip fractures^11,12^. However, several studies have associated BPs therapy with a potential risk of osteonecrosis of the jaw^13,14^ and atypical subtrochanteric femoral fractures; at the cellular level, prolonged exposure to BPs was shown to eventually cause the suppression of osteoblast function both by direct and OC-mediated mechanisms^15^. The inability to restore the lost bone structure may be the cause of poor bone quality and increased risk of atypical bone fractures in BPs treated patients^16^. Although the prevalence of side effect from BPs is very low, the impact from media reports has led to a dramatic decrease in the compliance to BPs therapies; for example, the use of alendronate (AL) has declined by over 50% from 2008 to 2012^17^. As a consequence, drugs capable of stimulating bone formation while maintaining anti-resorptive activity can provide strong advantages as compared to current BPs.

A growing body of evidence indicates that hydrogen sulfide (H_2_S) is a gaseous molecule produced in substantial amounts by mammalian tissues, which exerts a variety of physiological effects in different systems, including bone^18^. Consistently, the discovery of suitable H_2_S-donor agents and H_2_S-releasing multi-target drugs is currently considered a timely and challenging field of research in drug discovery^19^.

In particular, our group and others recently described that H_2_S inhibits h-OCs development *in vitro* ^20^ and stimulates osteogenic differentiation of human mesenchymal stromal cells (h-MSCs) *in vitro* and *in vivo* ^18,21^. Based on the dual action exerted by H_2_S, we sought to exploit these findings through the development of an H_2_S-releasing BPs, prototype of a novel family of hybrid molecules aiming at treating bone loss, by means of a multi-target pharmacological approach. An H_2_S-releasing BPs could allow a local delivery of H_2_S at the site where new bone formation is required thereby maximizing the osteogenic properties of H_2_S.

Ideal H_2_S-releasing moieties should generate H_2_S with a relatively slow rate; moreover, they should be stable, allowing an easy chemical manipulation^22^. In this perspective, we have recently investigated several organic sulfur compounds of either synthetic or natural origin^23,24^ and identified aryl-isothiocyanate as a suitable moiety, useful for building H_2_S-releasing hybrid drugs^25^.

This paper describes an innovative molecule, named DM-22, derived from the widely known N-BPs, AL, hybridized with an aryl-isothiocyanate - based H_2_S-releasing moiety. We investigated *in vitro* the potential beneficial effects on bone cells of DM-22 compared to that of the parent drug AL.

## Methods

### Chemical synthesis

Melting points were determined on a Kofler apparatus and are uncorrected. Chemical shifts (δ) are reported in parts per million and are calibrated using residual undeuterated solvent as an internal reference. 1H NMR,^31^P NMR and^13^C NMR spectra of all compounds were recorded with a Varian Gemini 200 spectrometer operating at 200 MHz or Bruker TopSpin 3.2 spectrometer operating at 400 MHz, in a ~2% solution of deuterated water (D_2_O), unless otherwise stated. Data for^1^H NMR spectra are reported as follows: chemical shift (δ ppm) (multiplicity, coupling constant (Hz), integration). Multiplicities are reported as follows: s = singlet, d = doublet, t = triplet, q = quartet, m = multiplet, br = broad, or combinations thereof. The 95% purity of tested compounds was confirmed by combustion analysis. ESI-MS/MS experiment was performed in negative ion mode, using a LCQ Advantage ion trap mass spectrometer (ThermoFinnigan, San Jose, CA, USA) equipped with Xcalibur 3.1 software. Merck gel plates (60 F254) were used for analytical TLC. UV light was used to examine the spots. Evaporation was performed in vacuo (rotating evaporator). Sodium sulfate was used as the drying agent. Commercially available chemicals were purchased from Sigma-Aldrich and TCI Chemicals. The synthesis of DM-22 was performed in 3 steps characterized by the preparation of the following intermediate products.

### 4-(4-nitrobenzamido)butanoic acid (1)

*p-*nitrobenzoic acid (300 mg, 1.64 mmol) was treated with thionyl chloride (685 mg, 5.76 mmol) at 80 °C for 12 h. The solvent was evaporated to yield a yellow solid which was dissolved in a small amount of THF and added dropwise to a solution of γ-aminobutyric acid (78 mg, 0.76 mmol) in aqueous NaOH (156 mg, 4.40 mL). The resulting solution was stirred at room temperature (RT) for 12 h; Then, the pH value was adjusted to 2 by the addition of HCl 1 N. The precipitate was collected and crystallized from H_2_O thus yielding a white solid corresponding to the product (**1)** (197 mg, yield 74%): ^1^H-NMR (CD_3_OD): δ 1.86–2.00 (m, 2 H, CH_2_); 2.41 (t, 2 H, *J* = 7.3 Hz, CH_2_); 3.46 (t, 2 H, *J* = 6.9 Hz, CH_2_), 8.02 (d, 2 H, J = 9.0 Hz, Ar); 8.32 (d, 2 H, J = 9.0 Hz, Ar) ppm.

### 4-(4-aminobenzamido)butanoic acid (2)

To a solution of 4-(4-nitrobenzamido)butanoic acid (77 mg, 0.31 mmol) in MeOH (2 mL) was added carbon (16 mg), a catalytic amount of FeCl_3_ and hydrazine monohydrate (116 mg, 5.18 mmol). The resulting mixture was refluxed for 24 h. Afterwards, the reaction mixture passed through a pad of celite and the solvent evaporated affording a crude product that was purified by precipitation from EtOH/Et_2_O (43 mg, yield 64%).^1^H-NMR (CD_3_OD): δ 1.80–1.94 (m, 2 H, CH_2_); 2.28 (t, 2 H, *J* = 7.2 Hz, CH_2_); 3.37 (t, 2 H, *J* = 6.8 Hz, CH_2_), 6.66 (d, 2 H, *J* = 8.5 Hz, Ar); 7.60 (d, 2 H, *J* = 8.5 Hz, Ar) ppm.

### (1-hydroxy-4-(4-isothiocyanatobenzamido)butane-1,1-diyl)diphosphonic acid (DM-22)

A solution of cathecol borane in THF 1 M (0.88 mL, 0.88 mmol) was added to compound 2 (63 mg; 0.28 mmol) under N_2_ atmosphere. The reaction mixture was stirred at RT for 1 h. Then, P(OSiMe_3_)_3_ (347 mg, 1.16 mmol) was added to the reaction mixture and stirred at rt for 16 h. The solvent was removed in vacuo to give a crude residue, which was triturated with CHCl_3_. The crude oil was dissolved in a solution of NaHCO_3_ 0.4 M (2 mL) and thiophosgene (228 mg, 1.98 mmol) was added to the reaction mixture and stirred at RT for 2 h. Finally, the reaction mixture was extracted with dichloromethane (DCM). The combined organic layers were dried over MgSO_4_, and filtered, and the solvent was removed in vacuo to give a residue, which was triturated with hexane to obtain the final product (20 mg, yield 24%).^1^H-NMR (D_2_O): δ 1.90–2.15 (m, 4 H, CH_2_); 3.45 (t, 2 H, *J* = 6.6 Hz, CH_2_); 7.43 (d, 2 H, *J* = 8.8 Hz, Ar), 7.80 (d, 2 H, *J* = 8.8 Hz, Ar) ppm.^13^C-NMR (D_2_O): δ 167.70, 129.37, 128.93, 126.39, 124.06, 123.70, 76.66, 40.56, 31.24, 23.52 ppm.^31^P- NMR (D_2_O) δ 19.23 ppm; ESI-MS m/z calculated for C_12_H_16_N_2_O_8_P_2_S, 410.01; found, 409.45. [M - H^+^].

### Determination of H2S release by amperometric assay

The characterization of the potential H_2_S-generating properties of DM-22 has been carried out by an amperometric approach, through the Apollo-4000 free radical analyzer (WPI) detector and H_2_S-selective mini-electrodes. 6 different recordings have been carried out at RT. Following the manufacturer’s instructions, a “PBS buffer 10x” was prepared (NaH_2_PO_4_•H_2_O 1.28 g, Na_2_HPO_4_.12H_2_O 5.97 g, NaCl 43.88 g in 500 ml H_2_O) and stocked at 4 °C. Immediately before the experiments, the “PBS buffer 10x” was diluted using distilled water (1:10) to obtain the assay buffer and the pH adjusted to 7.4. The H_2_S-selective mini-electrode was equilibrated in 10 ml of the assay buffer, until the recovery of a stable baseline. Then, 100 μl of a dimethyl sulfoxide (DMSO) solution of DM-22 was added (the final concentration of DM-22 was 1 mM; the final concentration of DMSO in the assay buffer was 1%). The generation of H_2_S was observed for 20 min. Preliminary experiments demonstrated that DMSO 1% did not produce any interference on the amperometric recording. When required by the experimental protocol, L-Cysteine (final concentration 4 mM) was added 10 min before the addition of DM-22. L-Cysteine alone did not produce any amperometric response. The correct relationship between the amperometric currents (recorded in pA) and the corresponding concentrations of H_2_S was previously determined by suitable calibration curves, which were obtained by the use of sodium hydrosulfide, an H_2_S donor, (NaHS; 1-3-5-10 μM) at pH 4.0.

### h-monocytes (CD11b^+^ cells) isolation

In preparing samples for human tissues, all protocol and procedures were in accordance with the ethical standards of the institutional ethical committee of the Rizzoli Orthopedic Institute, who approved the study, and with the 1964 Helsinki Declaration and its later amendments.

Human monocytes (h-monocytes) were isolated from peripheral blood mononuclear cells (PBMCs) of 3 healthy donors, carrying out a gradient separation with Ficoll (Lympholite-H, Cederlane, Burlington, Ontario, Canada) followed by immunomagnetic positive selection of CD11b^+^ cells (MACS system, Miltenyi Biotech, Calderara di Reno, Italy), according to procedures well established in our laboratory^26^. Purity of CD11b^+^ enriched population was assayed by flow cytometry (FACS) analysis using FACS canto II (BD bioscience, San Jose, California, USA).

### h-OCs differentiation

h-monocytes were seeded in quadruplicate into 96 well-plates at a density of 5 × 10^5^ cells/cm^2^. h-OCs were obtained by culturing h-monocytes for 6 days α–MEM medium (Euroclone, Milan, Italy) supplemented with 10% FBS (Lonza, Basel, Switzerland) and 1% penicillin/streptomycin, in the presence of M-CSF (10 ng/ml) and RANKL (75 ng/ml) (Miltenyi Biotech). Cells were cultured in presence or absence of AL and DM-22 (1, 3.3, 10, 33 µM); medium and stimuli were replaced three times *per* week.

### Cytotoxicity and TRAP assay on OCs

h-monocytes were seeded as above and cultured in α–MEM 5% FBS (Thermo Fischer Scientific, Waltham, Massachusetts, U.S.A) depleted of phenol-red for 24–72 h in the presence or absence of increasing concentrations of AL and DM-22 (1-3.3-10-33 µM). Lactate dehydrogenase (LDH) assays (Cytotoxicity detection kit (LDH), Roche) were performed on supernatants, according to manufacturer instructions, to test the acute toxicity. Colorimetric detection of LDH was performed at 492–620 nm on TECAN instrument and cytoxicity calculated with reference to the control (unstimulated samples) and positive control (Triton X-100 treated samples) according to the formula: [(experimental value-unstimulated control value)/(Positive control-unstimulated control value)]*100. According to the previous formula unstimulated control value express a 0% of cytotoxicity while positive control a 100% of cytotoxicity.

To evaluate OC differentiation, tartrate acid phosphatase (TRAP) assay (Acid Phosphatase, Leukocyte (TRAP) Kit, Sigma Aldrich) was performed after 7 days in culture. Mature h-OCs were defined as TRAP positive cells containing at least three nuclei and were manually counted using an inverted microscope; 8 microscope fields at 20X magnification were counted for each duplicate well and the h-OCs count was expressed as average h-OCs number/fields.

### Functional assay for mature h-OCs (pit assay)

For pit assays, h-monocytes were seeded on synthetic hydroxyapatite-coated 16-well slides (Osteologic slides, BD Pharmingen, Franklin Lakes, NJ, USA), which mimics *in vitro* bone matrix, at the density of 5 × 10^5^/cm^2^.

h-OCs were obtained by culturing h-monocytes for 7 days in osteoclastogenic medium in presence or absence of AL and DM-22 (1–3.3–10–33 µM). After washing with bleach to eliminate cells, pits formed on the Osteologic slides upon matrix breakdown by h-OCs, were captured by NIS software (Nikon, Firenze, Italy) and Nikon Instruments Europe BV (Amstelveen, the Netherlands) using differential interference contrast system and evaluated.

### h-MSCs isolation and culture

Bone resident h-MSCs were isolated, after obtaining informed consent by each donor, from the tybial plateau of 6 patients undergoing surgical knee replacement according to procedure well established by our laboratory^27^. Briefly, bone fragments were mechanically fragmented into small pieces to generate a cell suspension which was subjected to Ficoll-density gradient isolation protocol as previously reported^27^. Cells were grown and expanded in α–MEM medium supplemented with 15% FBS and 1% penicillin/streptomycin until passage 2.

### Cytotoxicity assay on hMSCs

h-MSCs were seeded into 96 well-plates at a concentration of 3 × 10^4^ cells/ cm^2^ in α–MEM 7,5% FBS depleted of phenol-red for 24–72 h in the presence or absence of increasing concentrations of AL and DM-22 (1–3.3–10–33 µM). Morphological analysis was examined under light microscope. Photos were taken using Nikon Instruments after having performed toluidine blue assay for increasing the sharpness of cells. Cells were fixed in formalin 10% for 20 min, toluidine was added for few seconds and then cells were washed with deionized water to eliminate unspecific staining. Afterwards, the positive staining was measured at 560 nm on TECAN Infinite® 200 PRO (Tecan Italia S.r.l., Cernusco Sul Naviglio, Italy). LDH assays (Cytotoxicity detection kit (LDH), Roche) were performed as described above.

### h-MSCs proliferation

h-MSCs were seeded in quadruplicates in 96-well plates at 3 × 10^4^ cells/cm^2^ and cultured in α-MEM 15% FBS at 37 °C, 5% CO_2_ and 95% O_2._ Proliferation was tested after 24 and 72 h in culture. 18 h before each time point, 5 µCi of^3^H-thymidine (Perkin Elmer, Boston, MA, USA) was added to each well and radioactivity was then measured using a beta-counter (Perkin Elmer).

### h-MSCs osteogenic differentiation

Cells were harvested and seeded into 12 well-plates at a concentration of 5 × 10^4^ hMSCs/cm^2^. Cells were induced toward osteogenic differentiation with α-MEM 20% FBS supplemented with 0,1 µM dexamethasone (Sigma Aldrich, St. Louis, MO, USA), 100 µM ascorbic acid (Sigma Aldrich) and 10 mM β-glicerolphosphate (Sigma Aldrich) in the presence or absence of AL and DM-22 (1–3.3–10–33 µM). Cells were cultured for up to 21 days at 37 °C, 5% CO_2_ and 95% O_2_ and medium was replaced twice *per* week. Alizarin Red S (AR-S) staining (Sigma Aldrich) was performed as follows. Cells were fixed for 15 minutes at RT in formaldehyde (Kaltek, Padova, Italy) 10% phosphate buffered saline (PBS), washed twice with PBS, stained with 40 mM AR-S for 20 minutes at RT and washed with deionized water to eliminate unspecific staining. A spectrophotometric analysis with TECAN was used to quantify AR-S positive stain. Briefly, absorbance was red at 510 nm, as previously detailed^27^, and data were further elaborated subtracting the levels of background. As a result, readings which do not detect a positive stain gave negative values.

### Reverse Transcription-Polymerase chain reaction (RT-PCR)

To evaluate gene expression, RNA was isolated with RNApure (Euroclone), and purified from DNA with DNA-free™ Kit (Ambion, Life Technologies; Carlsbad, California, U.S.A) according to manufacturer instructions. RNA concentration was assessed by Nanodrop 2000c (Thermo Scientific, Rockford, IL, USA) and the quality of RNA was analyzed through the 260/280 and 260/230 nm absorbance ratio. Only samples showing a 260/280 ratio >1.8 were used for the following transcription step. Reverse transcription (SuperScript® *VILO*™ cDNA Synthesis Kit; Invitrogen, Life Technologies) was performed utilizing 0,5-1 µg of RNA according to manufacturer instruction as follows: 25 °C for 10 minutes, 42 °C for 60 minutes, 85 °C for 5 minutes and 4 °C for 30 minutes on 2720 Thermal cycler (Applied Biosystem, Life Technologies). Polymerase chain reaction (PCR) (SYBR Premix Ex Taq, TaKaRa Biomedicals, Tokyo, Japan; LightCycler Instrument, Roche) was performed on 20 ng cDNA as following: one cycle at 95 °C for 10 seconds and 45 cycles at 95 °C for 5 seconds and at 60 °C for 20 seconds. The specificity of the PCR products was confirmed by standard melting curve analysis with the following thermal cycling profile: 95 °C for 10 seconds, 65 °C for 15 seconds and 95 °C in one-degree increments. Relative quantification of PCR products was obtained with the comparative C_T_ method, comparing to the housekeeping mRNA expression of glyceraldehyde-3 phosphate dehydrogenase (GAPDH). All the primers, whom sequences are reported in Table 1, were obtained from Life Technologies.

**Table 1:** Primers sequences utilized in the experiments.

Extended name	GENE symbol		5′-Sequence-3′	Product size (bp)	Accession number
Glyceraldehyde-3 phosphate dehydrogenase	GAPDH	Forward	CGGAGTCAACGGATTTGG	218	NM_002046
		Reverse	CCTGGAAGATGGTGATGG		
Alkaline phosphatase	ALP	Forward	GGAAGACACTCTGACCGT	152	NM_000478
		Reverse	GCC CAT TGC CAT ACA GGA		
Bone sialoprotein	BSP	Forward	CAGTAGTGACTCATCCGAAG	158	NM_004967
		Reverse	CATAGCCCAGTGTTGTAGCA		
Collagen type I	COLL I	Forward	GAGAGCATGACCGATGG	251	NM_000088
		Reverse	GTGACGCTGTAGGTGAA		
Collagen type XV	COLL XV	Forward	AAGCCGTCACCTACACTCAA	228	NM_001855
		Reverse	CACCATCCACAGAATGAACC		

## Statistical analysis

GraphPad Prism 7 (La Jolla, CA, USA) and IBM SPSS Statistics (New York, USA) software were used for statistical analyses. D’Agostino-Pearson omnibus test was performed to test normality of continuous variables. Data on H_2_S release by DM-22 were analyzed by two-way ANOVA followed by Bonferroni post-test.

Multiple comparisons were made by one-way ANOVA and Tukey multiple comparison tests or Kruskall-Wallis and Dunn’s multiple comparison test, respectively in parametric or non-parametric data set. Simple comparisons were made by using Unpaired t-test or Mann-Whitney test, respectively in parametric or non-parametric data set. Wilcoxon signed rank test was used to compare each sample with a hypothetical value (1 or 100) for analysis of fold increase. A statistical multivariate analysis, the Generalized Linear Model (GLM), was used to analyze three groups of non-parametric data in the osteogenic gene expression data set.

P values < 0.05 were considered statistically significant.

## Data Availability

The datasets generated during and/or analyzed during the current study are available from the corresponding author on reasonable request.

## Results

### Chemistry

**Figure 1:**
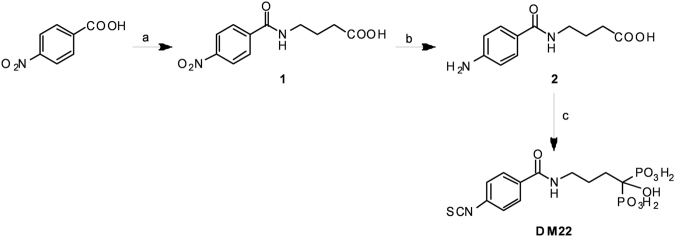
Scheme of chemical synthesis of DM-22. Reagents and conditions**:** a) (i) SOCl_2_, 80 °C, 2 h (ii) γ-aminobutyrric acid, NaOH, 5 °C → RT 12 h; b) NH_2_NH_2_, FeCl_3_, C, MeOH, 24 h; c) (i) catecholborane 1 M, P(OSiMe_3_)_3_, 1 h (ii) thiophosgene, NaHCO_3_ 0.4 M, 2 h.

The new hybrid compound was synthesised as reported in Fig. 1. Briefly, the condensation of p-nitrobenzoic acid with the γ-aminobutirric acid in an aqueos solution of NaOH yielded the amide **1** (Fig. 1a). The subsequent reduction of the nitro group with hydrazine monohydrate in the presence of FeCl_3_ gave the 4-(4-aminobenzamido)butanoic acid **2** (Fig. 1b). The intermediate namely the 1-hydroxy-1,1,-bisphosphonic acid was obtained through an efficient and simple one-pot synthesis starting from the carboxylic acid **1**, cathecol-borane and tris(triethylsylilphosphate)^28^. The final reaction with thiophosgene and NaHCO_3_ yielded the isothiocianate derivative DM-22 (Fig. 1c). The H_2_S donor-BP was characterized by ^1^H, ^13^C, ^31^P NMR spectra.

### DM-22 is a slow-releasing H2S donor

**Figure 2:**
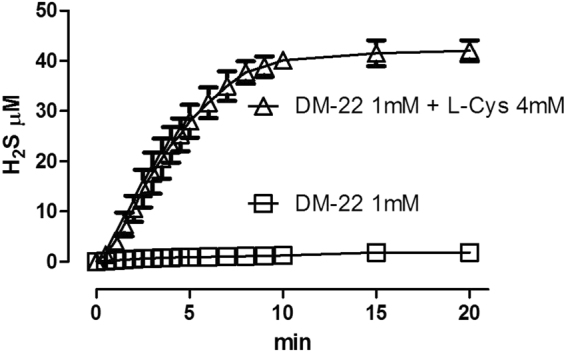
Graph of H_2_S release from DM-22. The graph shows the progressive increase of the concentration of H_2_S, following the addition of DM-22 into an acqueous solution (pH 7.4) in the absence (squares) or in the presence (triangles) of an excess (4 mM) of L-Cysteine. Results are expressed as mean ± SEM of 6 different recordings.

The incubation of DM-22 1 mM in aqueous solution (assay buffer, at pH 7.4 and 20 °C), in the presence of L-Cysteine 4 mM, led to the generation of a time-related increasing concentration of H_2_S (Fig. 2). The rise of the H_2_S production reached a steady-state after about 10 min and the highest concentration of H_2_S, recorded after 20 min was 42.0 ± 2.1 µM. In contrast, in the absence of L-Cysteine, the incubation of DM-22 1 mM led to the formation of dramatically lower levels of H_2_S (1.8 ± 0.1 µM, after 20 min of incubation). Such a organic thiol-dependent mechanism of H_2_S release is in agreement with previous observations on aryl-isothiocyanates^25^, and even on different chemotypes of H_2_S-donors, such as thioamides^23^ or organic disulfides^29^.

### DM-22 inhibits h-OCs differentiation and function without inducing cytotoxicity

**Figure 3:**
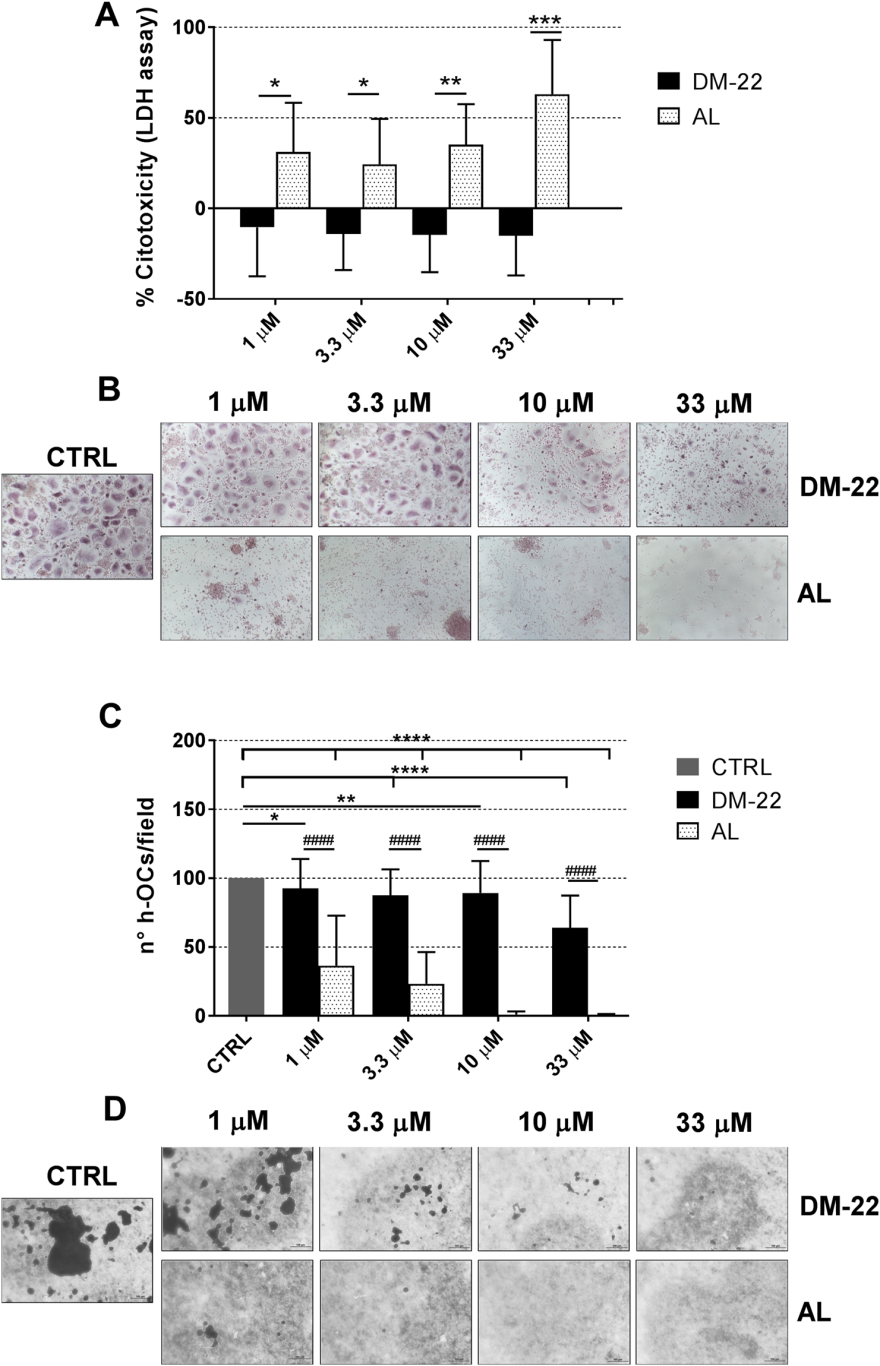
DM-22 inhibits h-OCs differentiation and function without inducing cytotoxicity and preserving a residual h-OCs population. (**A)** Histogram showing LDH measurements. Data are expressed as percent cytotoxicity and refers to arbitrary units obtained by colorimetric detection of LDH activity. Data are expressed as mean ± SD of triplicates of N = 3 independent experiments. One sample t-test was performed for statistical analyses (*p < 0.05, **p < 0.01, ***p < 0.001 DM-22 *vs* AL). (**B**) Representative pictures of TRAP staining (magnification 20X), showing the effect of DM-22 and AL on h-OCs differentiation. (**C**) Histograms showing the average number of TRAP^+^ h-OCs per microscope field. At least 8 fields were counted for each well in duplicate. Data are expressed as mean ± SD of N = 3 independent experiments. Mann-Whitney test (^####^p < 0.0001 DM-22 *vs* AL) or Wilcokon signed rank test (*p < 0.05, **p < 0.01, ****p < 0.0001 *vs* CTRL = 100%) were performed for statystical analyses. (**D**) Representative pictures of pit assay (magnification 20X), showing the effect of DM-22 and AL on h-OCs function.

Firstly we investigated the safety profile of DM-22 by analyzing its cytotoxicity on mature h-OCs by LDH assay. As shown in Fig. 3A, DM-22 displayed no cytotoxicity throughout the concentration range tested. Negative values of cytotoxicity are explained by the fact that DM-22 stimulation resulted in reading values below the baseline levels generated by control, unstimulated sample (=0). On the contrary, AL consistently revealed a detectable cytotoxicity, which was significantly higher than DM-22 at each concentration tested in our experiments (p < 0.05 for 1 and 3.3 μM, p < 0.01 for 10 μM, p < 0.0001 for 33 μM versus DM-22, Fig. 3A).

Next, we asked whether DM-22 retained the AL-like inhibition on osteoclastogenesis. Analysis of osteoclastogenic differentiation revealed a different pattern of inhibition by DM-22 compared to AL. Representative pictures of TRAP ^+^ hOCs at the end of the culture period are shown in Fig. 3B. DM-22 significantly decreased the number of mature hOCs at all the concentrations tested (Fig. 3C; p < 0.05 for 1 μM, p < 0.01 for 3.3 and 10 μM, p < 0.0001 for 33 μM *vs* control sample). Inhibition of h-OCs differentiation peaked at the high concentration of 33 μM, where no cytotoxicity had been shown, and reached nearly 40% inhibition relative to control samples.

As expected, AL dose-dependently decreased the total number of TRAP^+^ h-OCs, resulting into highly significant inhibition at all the concentrations tested (p < 0.0001 *vs* control sample) (Fig. 3C). In particular AL decreased the number of h-OCs by 62% and 77%, respectively, at 1 and 3.3 μM and virtually abolished h-OCs differentiation starting from the concentration of 10 μM.

These data suggest that DM-22 likely preserve a residual population of h-OCs, important for the mantainment of bone turnover.

Coherently, the ability of mature h-OCs to break down a mineral substrate, as tested *in vitro* by the ‘pit assay’, was strongly prevented by 1 μM of AL treatments and virtually completely inhibited by higher concentrations (Fig. 3D). Conversely, h-OCs function was dose-dependently inhibited by DM-22.

### DM-22 is devoid of AL-like cytotoxicity on h-MSCs

**Figure 4:**
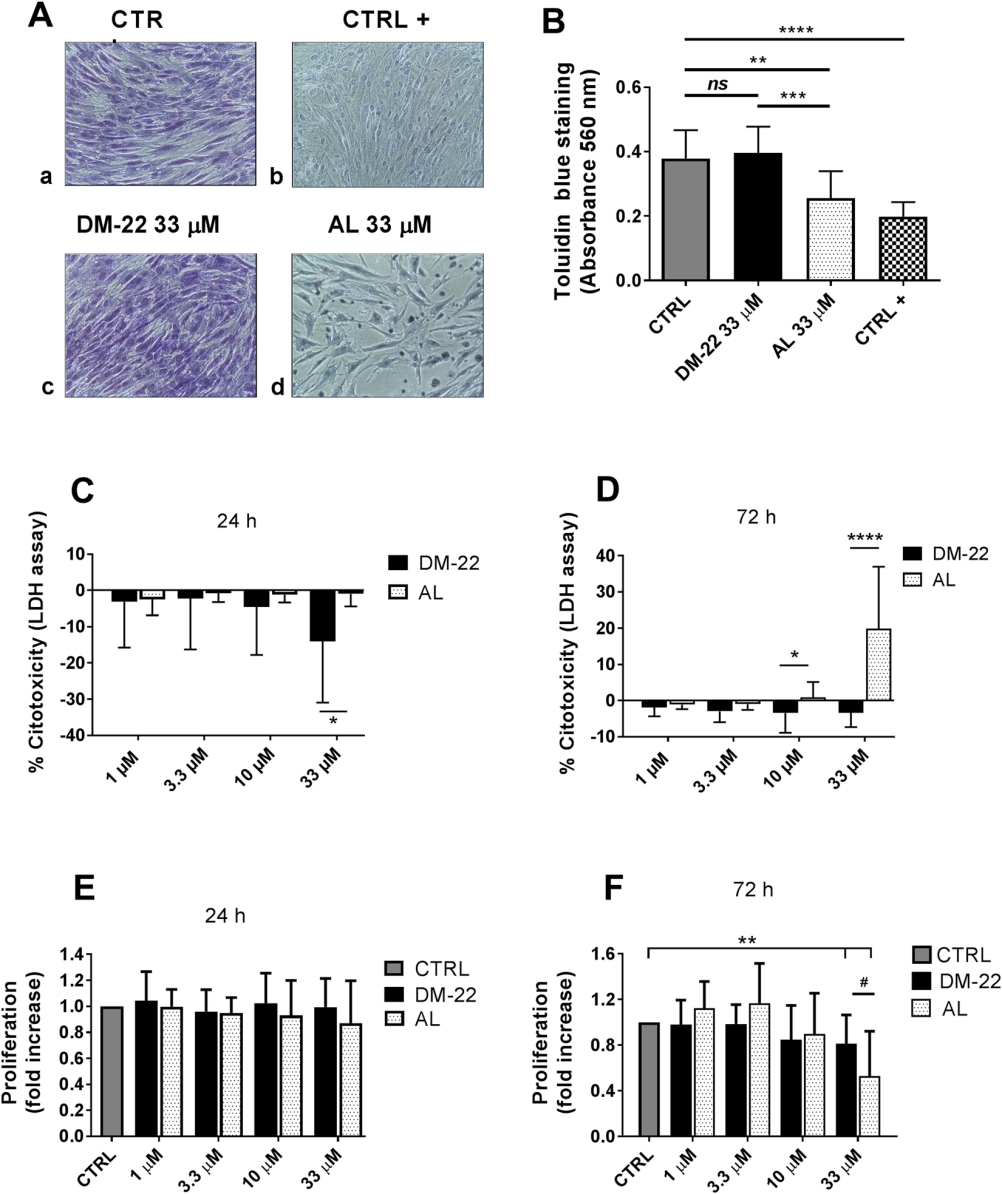
DM-22 is devoid of AL-like cytotoxicity on h-MSCs. (**A**) Representative pictures of toluidin blue staining showing the morphology of AL or DM-22 treated h-MSCs (33 μM) compared to untreated cells (CTRL) and cells treated with Triton X-100 (CTRL+). Magnification 20x. (**B**) Histogram showing toluidin blue staining quantification (absorbance 560 nm). Data are expressed as mean ± SD of N = 3 independent experiments (each one in quadruplicate). One way Anova and Tukey’s multiple comparison test were performed for statistical analyses. (**C**,**D**) Histograms showing LDH measurements in h-MSCs treated with DM-22 or AL compared to CTRL cells at 24 h (**C**) and 72 h (**D**); data are expressed as percent cytotoxicity and refers to arbitrary units obtained by colorimetric detection of LDH activity. Data are expressed as mean ± SD of N = 3 independent experiments (each one in quadruplicate). Mann Whitney test was performed for statistical analyses (*p < 0.05, ****p < 0.0001 DM-22 *vs* AL). (**E**,**F**) Histograms showing h-MSCs proliferation as revealed by 3H-thymidine detection for DM-22 and AL compared to CTRL cells at 24 h (**E**) and 72 h (**F**) Data are expressed as fold increase compared to CTRL sample and are expressed as mean ±SD of N = 4 independent experiments (each one in quadruplicate). Wilcoxon signed rank test (*p < 0.05, **p < 0.01) and Mann-Whitney test (^#^p = 0.0516) were performed for statistical analyses.

Next we investigated the effect of DM-22 on h-MSCs on viability and proliferation. Staining with the vital dye toluidine blue revealed that treatment with DM-22 and AL at the highest concentration (33 μM) triggered a different response on h-MSCs after 72 h in culture (Fig. 4A,B). While DM-22 did not alter the intensity of staining compared to control samples (Fig. 4A-a,c; Fig. 4B), AL induced a marked decreased in cellularity (Fig. 4A-a,d); moreover, after AL treatment, h-MSCs showed occasionally picnotic nuclei (Fig. 4A-d) and the intensity of staining was significantly decreased compared to control samples, close to the value induced by treatment with the detergent Triton X-100 (CTRL+, Fig. 4B).

Next we investigated the acute cytotoxicity within a time-range of 72 h. Quantification of LDH assay performed after 24 h (Fig. 4C) and 72 h (Fig. 4D) confirmed that DM-22 was completely devoid of cytoxicity at all the concentration tested. Consistant with data revealed by toluidine blu staining, the highest concentration of AL resulted in significant cytoxicity in h-MSCs after 72 h in culture (Fig. 4D; 20%; p < 0.0001). Significant cytotoxicity was also induced by 10 μM AL, however the percentage of inhibition is negligible compared to that of the highest dose (Fig. 4D; 1%; p < 0.05).

Analysis of cell proliferation in h-MSCs also revealed that AL treatment induce a dose-dependent decrease in cellular proliferation, resulting into a significant inhibition at 33 μM at 72 h in culture (Fig. 4E,F). The highest concentration of AL induced a maximum inhibition of proliferation of nearly 50% relative to control samples after 72 h (p < 0.01). Although DM-22 also induced a significant inhibition of cell proliferation at the highest concentration (20%, p < 0.01), it showed a markedly lower inhibition of cell proliferation compared to AL (Fig. 4F; p < 0.05).

### DM-22 stimulates mineralization and expression of osteogenic markers in h-MSCs

**Figure 5:**
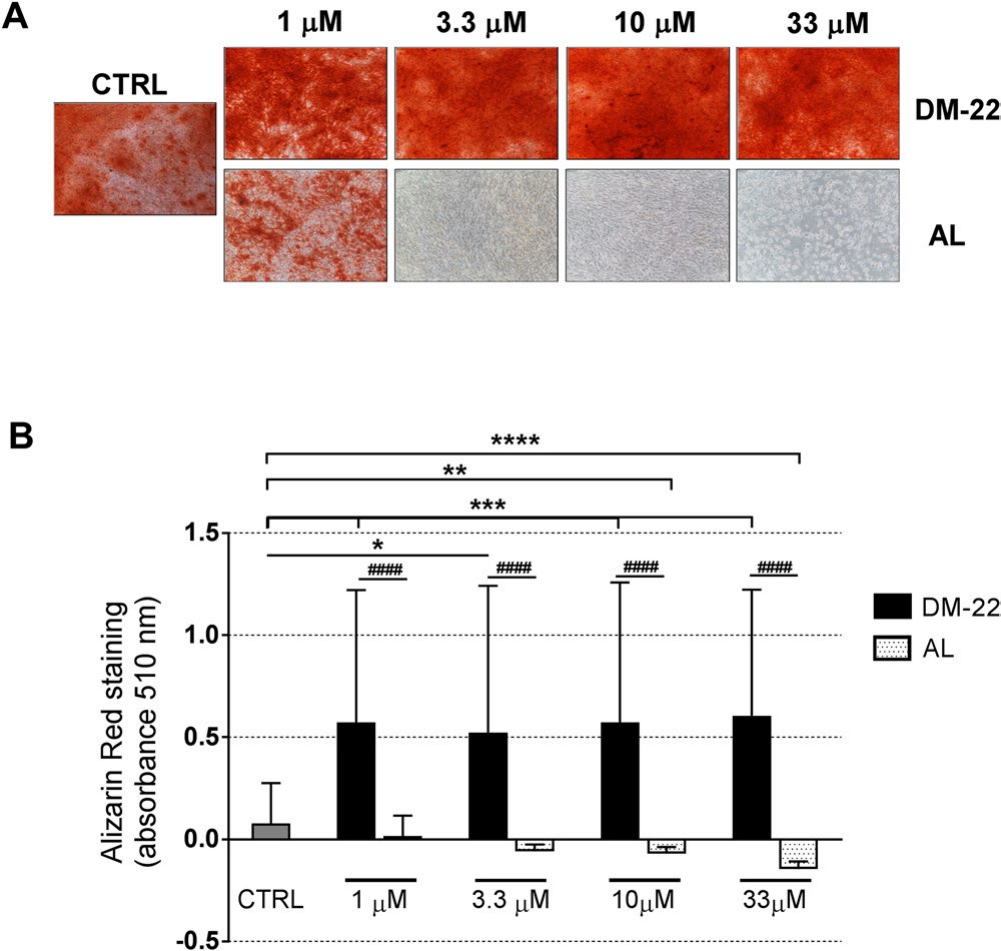
DM-22 increased mineralization in osteogenic h-MSCs. (**A**) Representative pictures of AR-S staining showing the effect of DM-22 and AL on h-MSCs differentiation. Magnification 20X. (**B**) Histogram showing AR-S staining quantification. Data are expressed as mean ± SD of N = 8 independent experiments. Kruskal-Wallis +Dunn’s multiple comparison test (*p < 0.05, **p < 0.01 ***p < 0.001, ****p < 0.0001) and Mann Whitney test (^####^p < 0.0001) were used for statistical analyses.

To further assess the effect of DM-22 on h-MSCs function, we investigated bone resident h-MSCs during *in vitro* osteogenic differentiation. Figure 5A shows representative pictures of AR-S staining at the day 21 time point for each treatment. As expected, control cultures of h-MSCs under osteogenic stimuli produce large mineralized nodules (Fig. 5A); interestingly, stimulation with DM-22 resulted in further increase of mineral matrix apposition, which induced a significant increase in mineralization as detected by quantification of nodules positive to AR-S staining at all the concentrations tested (Fig. 5B, p < 0.05, p < 0.001). A similar treatment with AL induced a marked inhibition of mineralization, which became evident by 1 μM and 3.3 μM (Fig. 5A), concentration levels that did not previously show cytoxicity (Fig. 4C,D). Consistant with findings showing rising cytoxicity of AL, stimulation with higher concentrations of AL resulted in the complete inhibition of mineral matrix deposition by h-MSCs (Fig. 5A,B; p < 0.01, p < 0.0001).

To further investigate the nature of DM-22 stimulation of osteogenic differentiation, we evaluated mRNA expression of osteogenic markers during the osteogenic culture (D14) and at the end of the experiment (D21).

**Figure 6:**
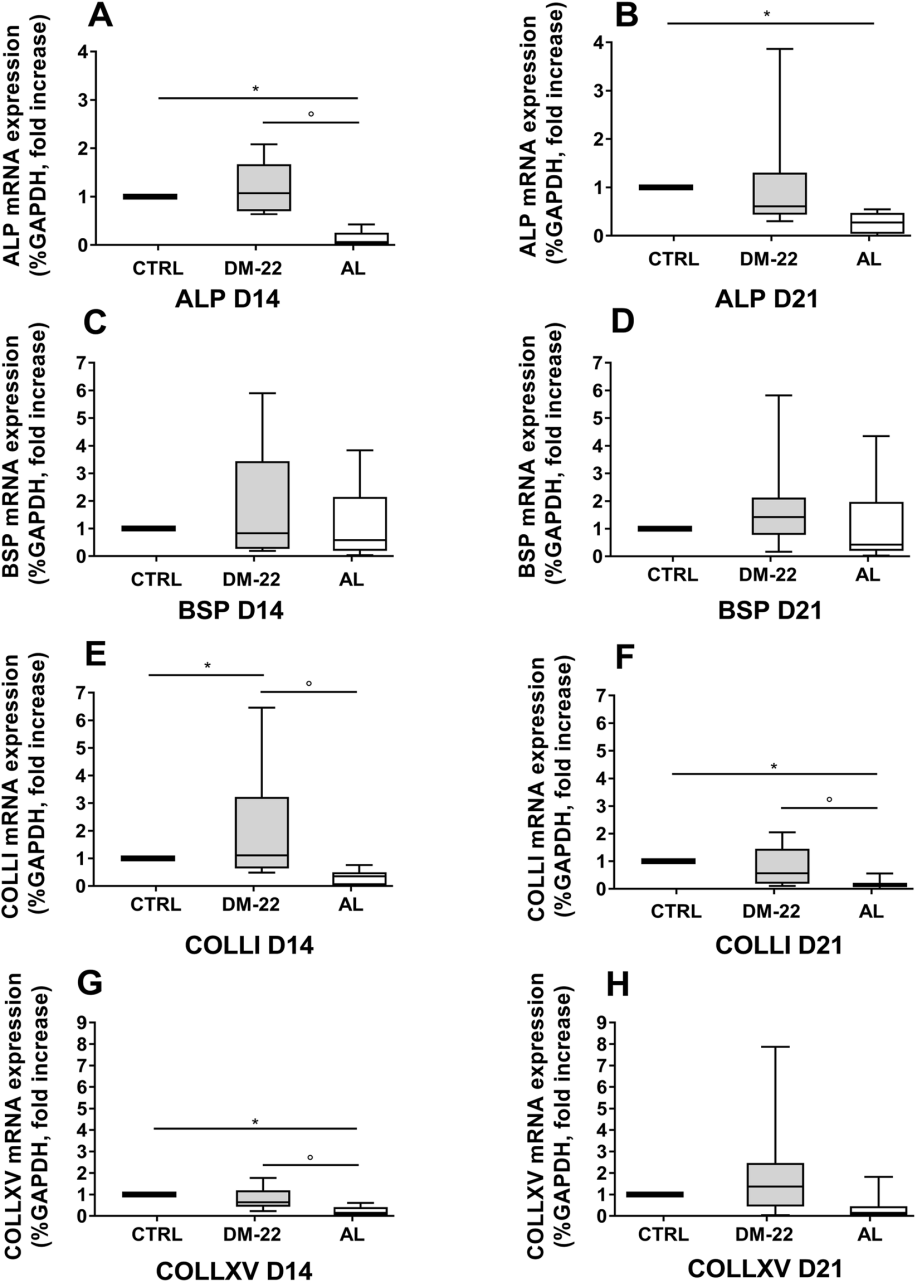
DM-22 stimulates gene expression of osteogenic markers. (**A**–**H**) Box plots showing mRNA expression of osteogenic markers in hMSCs after D14 (A,C,E,G) or D21 (B,D,F,H) in culture in the presence of DM-22 or AL. Expression levels of: ALP (**A**-**B**), BSP (**C**-**D**); COLL I (**E**,**F**); COLLXV (**G**,**H**) are reported. Data were analyzed by GLM analysis. Data are expressed as mean ± 95% confidence interval of fold increase compared to CTRL cells (=1) of N = 4 independent experiments. *p < 0.05 *vs* control samples; °p < 0.05 DM-22 versus AL.

Figure 6 summarizes gene expression of ALP (Fig. 6A,B), BSP (Fig. 6C,D), COLL I (Fig. 6E,F) and COLL XV (Fig. 6G,H). The GLM statistical analysis revealed that mRNA expression was affected by DM-22 or AL independent of the dose used; as a consequence, data are represented as box plots showing the overall effect of stimuli on the expression of target genes. h-MSCs stimulated with DM-22 showed significantly higher expression of COLLI at D14 in culture (Fig. 6E, p < 0.05) compared to control samples. DM-22 also induced gene expression above that of control samples for ALP at D14 (Fig. 6A), BSP at D21 (Fig. 6B), COLLXV at D21 (Fig. 6H); however, due to the high variability in response, these comparison did not achieve statistical significance in our analysis. When compared to AL-treated h-MSCs, DM-22 induced consistantly higher gene expression level for all the endpoints examined; in particular, mRNA of ALP D14 (Fig. 6A, p < 0.05), COLLI D14-D21 (Fig. 6E,F, p < 0.05) and COLLXV D14 (Fig. 6G, p < 0.05) was significanlty higher in samples treated with DM-22 compared to AL. Moreover, AL suppressed mRNA expression of ALP at D14-D21 (Fig. 6A,B, p < 0.05), COLLI at D21 (Fig. 6F, p < 0.05) and COLLXV D14 (Fig. 6G, p<0.05) compared to control h-MSCs. Notably, for the same genes at the same time point, DM-22 stimulation did not suppress mRNA expression levels compared to control samples.

## Discussion

H_2_S has recently shown an unexpected role in bone biology as a pivotal regulator of bone turnover. In the present study, we exploited the properites of H_2_S into a novel H_2_S-releasing BPs, named DM-22, obtained by “chemical hybridization” of AL, the lead compound in the N-BPs class, with an aryl-isothiocyanate-based H_2_S-releasing moiety. We investigated the main pharmacodynamic features of DM-22 *in vitro* and assessed its efficacy and safety in h-MSCs and h-OCs.

Electrochemical measurements demonstrated that DM-22 released H_2_S with a long-lasting kinetic, in line with the feature of isothiocyanate-based H_2_S-donor^23,24^. In designing this hybrid drug, such a pharmacological property was expected to improve the whole pharmacological profile and to attenuate the possible cytotoxicity of the BPs molecular portion.

DM-22 has been designed to meet the need for molecules showing improved efficacy and safety profile as compared to existing drugs for bone metabolism.

BPs are the first-line treatment for the therapy of several bone-wasting conditions^30^. However, long-term treatments with BPs are associated with an increased risk of developing adverse events; for example, epidemiological studies have established a compelling association between BPs potency, duration of treatment and the risk for developing osteonecrosis of the jaw^31^ as well as atypical, low energy fractures^32^. Long-term therapy with AL has been recently associated with the accumulation of microcracks in bone leading to reduced bone strenght in patients^33^, consistant with the hypothesis that excessive suppression of bone turnover can deteriorate bone quality^16^. Moreover, a recent report from the multidisciplinary task force of the American Society for Bone and Mineral Research (ASBMR) highlighted that, although the benefits of BPs treatment by far outweighs the risk of incurring in side effects, over 50% of patients failed to adhere to treatment longer than 12 months due, at least in part, to concerns about side effects.

DM-22 did not show cytoxicity in h-OCs at any of the concentrations tested. However, DM-22 induced a significant reduction of the number of mature h-OCs at all concentrations, although the inhibition was more pronounced at the highest concentration of 33 μM. By contrast, AL triggered a much more pronounced inhibition of h-OCs differentiation which was accompanied by an increased cytoxicity; consistant with previous reports^34,35^, AL completely abolished h-OCs differentiation and their ability to resorb mineral matrix in the concentration range between 1 and 33 μM.

Although we did not investigate the mechanism for the reduced anti-resorptive activity of DM-22, it is conceivable that the structural modification induced at the R2 side chain of AL is responsible for such a decreased activity. Indeed, it is known that increasing the distance between the nitrogen and the phosphonate group in N-BP results into a decreased ability to inhibit farnesyl diphosphate synthase (FPPS) in OCs^36^. Overall, DM-22 showed a moderate ability to act as anti-resorptive agent and mitigated the effects of AL by preserving h-OCs viability even in the upper concentration range tested in our experiments.

At the cellular level, our findings support the hypothesis that AL directly inhibits osteogenic differentiation and cause cytotoxicity in osteoprogenitor cells. Whether AL stimulate or inhibits osteogenic differentiation of MSCs *in vitro* is a controversial matter in previous reports and appears to be dependent on the protocol used; our data correlate with similar findings obtained using h-MSCs^37^ and with work by Orriss *et al*. showing cytotoxic inhibition of osteoblast proliferation and function in rats by N-BPs in the low micromolar concentration range^15^. Moreover, in broad agreement with our observations, it was reported that AL inhibits bone nodule formation at concentrations 10-fold lower than those required to induce apoptosis in rabbit calvarian osteoblast^38^. By contrast, AL was shown to stimulate osteogenic differentiation of h-MSCs^39^ peaking at 10 nM, a concentration 100-fold lower than the low-dose used in our experiments. One possible explanation for these diverging effects could be that AL triggers an hormetic dose-response during osteogenic differentiation of h-MSCs.

Compared to its parent molecule, DM-22 showed a substantially improved profile of safety and efficacy on h-MSCs induced to osteogenic differentiation within the same concentration range. DM-22 was devoid of cytotoxicity even at the high dose of 33 μM; even though a slight decrease in cell proliferation was induced by DM-22 at the high concentration of 33 μM, it did not affect cell viability and the ability of this compound to significantly increase the extent of mineralizing matrix as compared to control h-MSCs.

Semi-quantitative analysis of gene expression in h-MSCs provided further evidence of the potential osteoanabolic role of DM-22. h-MSCs stimulated with DM-22 showed consistantly higher mRNA expression than AL-stimulated samples across all the osteogenic targets and timepoints evaluated in our analysis. In particular the capacity of DM-22 to significantly stimulate COLL I expression marked a striking contrast with the effect induced by AL and may extend the applications of DM-22 to conditions characterized by defective synthesis of collagen.

Based on these data, DM-22 is the first N-BPs to display both anabolic and anti-resorptive functions in bone cells *in vitro*. Its biological activity on h-MSCs and h-OCs resembles that of H_2_S-releasing drugs previously tested by our group and others^18,20,21^. In particular, *in vivo* studies have ascertained that H_2_S-donors promote bone formation and prevent the bone wasting effect of estrogen deficiency in mice^18^, making it DM-22 a suitable candidate to achieve a long sought mitigation of the marked suppression of bone turnover exerted by traditional BPs.

Most BPs are characterized by poor bioavailability and very high affinity for the bone mineral matrix. Even tough further studies to test the *in vivo* bioavailability of DM-22 should be done in the near future, it is conceivable that the addition of aryl-isothiocianate moiety together with the “phosphonate” head of the molecule would lead to a local accumulation of DM-22 at the mineral bone surface, thus facilitating the local delivery of H_2_S. This would not only avoid potential systemic cytotoxicity but would locally replenish a molecule that is lowered in the pathological condition of bone loss due to osteoporosis^18^.

In conclusion, this work described the synthesis of a new H_2_S-hybrid N-BPs able to induce osteogenic differentiation of h-MSCs and, at the same time, retaining an anti-osteoclastogenic activity *in vitro*.

To the best of our knowledge, no other BPs elicit this combination of pharmacological effects in h-OCs and h-MSCs. The relevant features of the DM-22 is that it resulted from the winning conjugation of an anti-catabolic agent with a H_2_S-releasing moiety. The new multitasking N-BPs conjugate DM-22 may therefore pave the way to the development of novel therapies for bone loss providing new agents with a reduced toxicity profile.

Our work marks a significant expansion of H_2_S-based therapeutics, which stems from the recognition of the important role of H_2_S as a signaling molecule in several organs and tissues^19,40^, to the field of bone metabolism. This work will provide novel therapeutical opportunities in the field of bone loss therapies.
